# Endomyocardial biopsy guided by intracardiac echocardiography as a key step in intracardiac mass diagnosis 

**DOI:** 10.1186/s12872-018-0749-9

**Published:** 2018-01-30

**Authors:** Marco Zanobini, Antonio Dello Russo, Matteo Saccocci, Sergio Conti, Elisa De Camilli, Giulia Vettor, Valentina Catto, Maurizio Roberto, Cesare Fiorentini, Giuseppe Viale, Claudio Tondo, Michela Casella

**Affiliations:** 1 Cardiac Arrhythmia Research Center, Centro Cardiologico Monzino IRCCS, Milan, Italy; 2 Division of Pathology and Laboratory Medicine, European Institute of Oncology, Milan, Italy; 3 Department of Cardiology, Centro Cardiologico Monzino IRCCS, Milan, Italy; 4 Department of Cardiovascular Surgery, Centro Cardiologico Monzino IRCCS, Via Carlo Parea 4, 20138 Milan, Italy; 5 Department of Cardiovascular Sciences, University of Milan, Milan, Italy; 6 Department of Oncology and Haemato-oncology, University of Milan, Milan, Italy

**Keywords:** Intracardiac echocardiography, Endomyocardial biopsy, Right ventricle, Intracardiac mass

## Abstract

**Background:**

Based on a plenty of different applications, intracardiac echocardiography (ICE) is now a well-established technology in complex electrophysiological procedures. Recently, ICE has become the most widely used ultrasound-based imaging tool to guide diagnostic endomyocardial biopsy (EMB). EMB of cardiac mass guided by ICE is an interesting application of ICE. Allowing a correct positioning of the bioptome, ICE reduce the procedure-related risks and the need of a diagnostic open-chest procedure reserving the more invasive approach to selected cases.

**Case presentation:**

Hereby we report a case series of right ventricular masses in which the EMB was safely and effectively performed under ICE guidance giving essential information for planning the therapeutic strategy.

**Conclusions:**

The diagnosis of both metastatic and primary cardiac tumors relies on the histopathological analyses. The endomyocardial biopsy is a valuable tool for preoperative diagnosis and surgical planning of intracardiac masses suspected for tumors. In our experience, the use of ICE for right ventricle EMB of an intracardiac mass is an attractive modality thanks to the precise localization of the cardiac structures and the ability to guide bioptic withdrawal in the target area.

**Electronic supplementary material:**

The online version of this article (10.1186/s12872-018-0749-9) contains supplementary material, which is available to authorized users.

## Background

Intracardiac echocardiography (ICE) is a well-established imaging technology frequently used in complex electrophysiological procedures, such as atrial fibrillation and ventricular tachycardia ablation. As in other scenario, ICE can contribute in improving the efficacy and the safety of procedures. It allows a better definition of the anatomical structures involved in ablation procedures and permits a continuous monitoring of catheter-tissue contact avoiding procedure-related complications [1]. For these reasons, ICE is becoming the most widely used ultrasound imaging tool in diagnostic guidance endomyocardial biopsy (EMB). In addition, the use of ICE is an attractive modality also for EMB of intracardiac mass, in particular for right-sided structures. ICE-guided EMB allows a correct position of the bioptome in relation to the intracardiac mass permitting a reduction of procedure-related risks.

We describe three different cases of EMB of right ventricular masses performed under ICE guidance. In all cases, ICE imaging was performed using a 9F ViewFlex™ Xtra 64-element phased-array and 4-way steerable ultrasound catheter (St. Jude Medical, St. Paul, MN, USA) inserted via the right femoral vein or 8F Acunav 4-way steerable ultrasound catheter (Biosense Webster, Diamond Bar, CA, USA). The technique used by our operators to perform EMB guided by ICE is described in Additional file 1: Movie 1.

The study was conducted according to our Institutional Review Board and in adherence to the Declaration of Helsinki. A written informed consent was obtained from all patients or their closest relatives.

## Case presentation

### Case 1

**Fig. 1:**
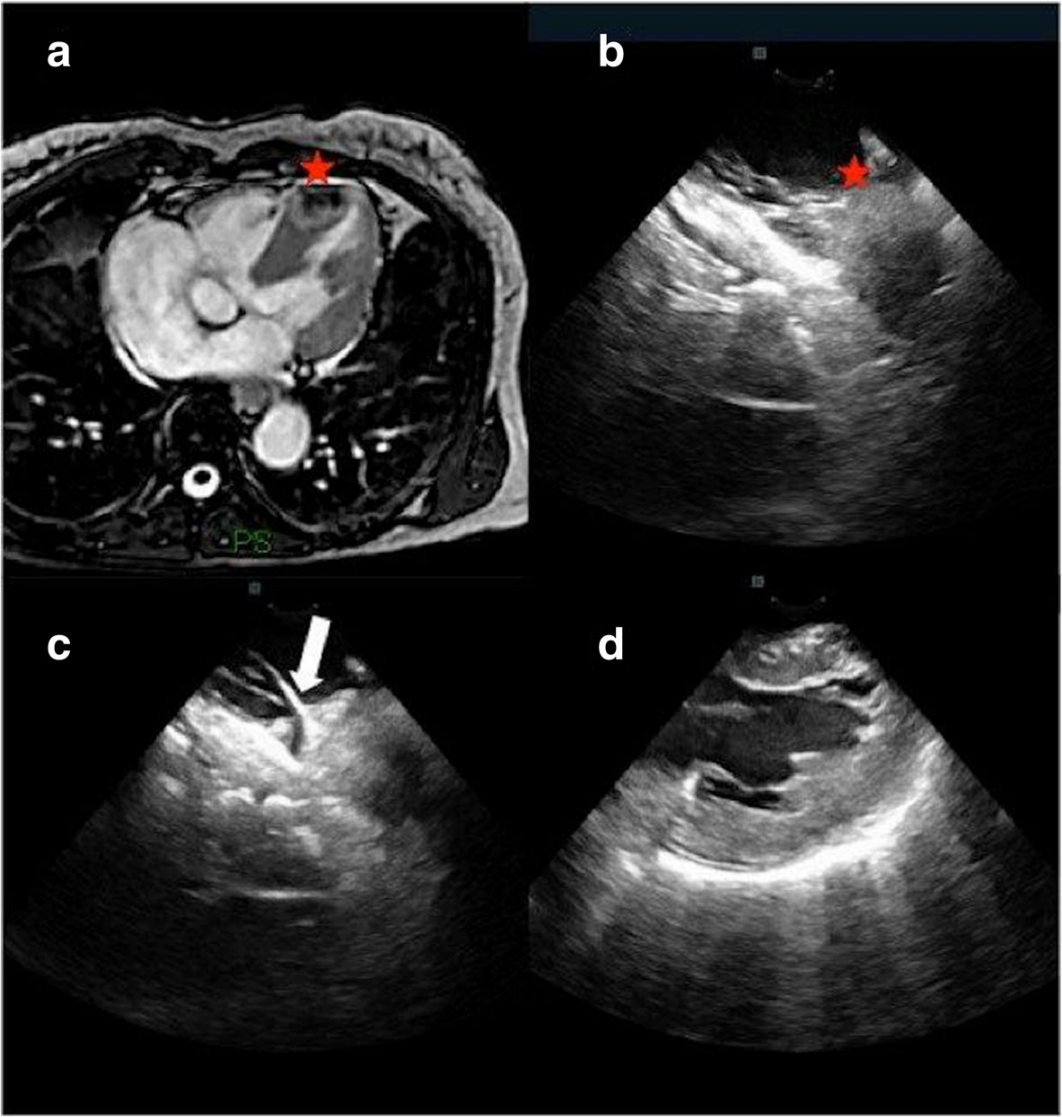
Cardiac magnetic resonance imaging: a 20 × 28 mm intramyocardial mass localized at the apex of the right ventricle (RV) is showed in panel (**a**). ICE shows the RV apical mass, panel (**b**). The bioptome (white arrow) is clearly visualized and is possible to confirm the good contact between the catheter and the mass, panel (**c**). After obtained all the biopsies needed, ICE is useful to exclude the presence of pericardial effusion, panel (**d**)

A 65-year-old woman smoker with a long history of malignancy was referred to our Center due to the evidence of an intracardiac mass at transthoracic echocardiography (TTE). Her past medical history included pulmonary adenocarcinoma treated with right lobectomy and lymphadenectomy, right nephrectomy due to clear cell renal carcinoma and total gastrectomy for adenocarcinoma. The TTE revealed a hyperechogenic thickening at the apex of the right ventricle (RV) without clear evidence of pathological mass. CMR showed a 20 × 28 mm intramyocardial mass localized at the apex of the RV (Fig. 1a). Due to previous history of malignancy there was a high suspicion of cardiac metastatic secondarism. Patient underwent percutaneous EMB of the RV mass under fluoroscopic and ICE guidance. The RV apical mass was visualized and five biopsies were performed (Fig. 1b and c; Additional file 2: Movie 2). No pericardial effusion was present at the end of the EMB (Fig. 1d). Pathologic specimens demonstrated no evidence of malignancy permitting to avoid open-chest surgical removal of the mass. The patient was safely discharged the day after EMB procedure and followed-up in outpatient clinic.

### Case 2

**Fig. 2:**
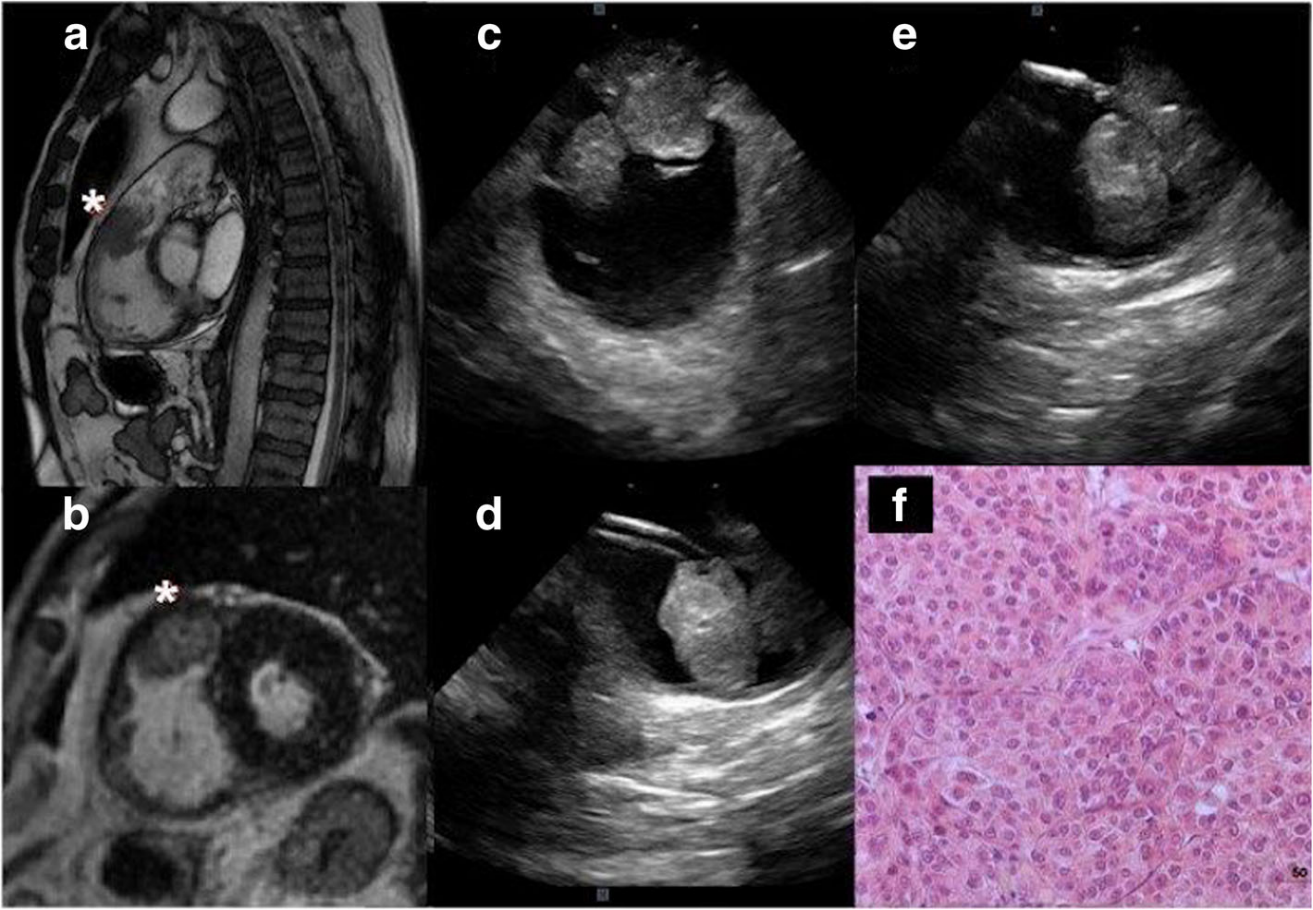
Cardiac magnetic resonance imaging: a rounded 30 × 17 mm mass based in the anterior portion of the right ventricular outflow tract (RVOT) involving the pulmonary valve is showed in panel (**a** and **b**) (white star). ICE clearly shows the mass, panel (**c**), and is useful to guide the correct positioning of the long-deflectable sheath through which the bioptome is introduced, panel (**d**). ICE visualizes a good contact between the bioptome and the mass, panel (**e**). Hystopathological diagnosis revealed a metastatic localization of hepatocellular carcinoma, panel (**f**)

A 54-years-old male, with previous history of liver transplant related to hepatocellular carcinoma (HCC) and HBV-cirrhosis, presented to our center for severe dyspnea. TTE demonstrated a 23 × 27 mm multi-lobed mass in the right ventricular outflow tract (RVOT) involving the pulmonary trunk. CMR imaging and contrast-enhanced CT also confirmed the intracardiac mass detected by TTE (Fig. 2a and b). Patient was scheduled for EMB under ICE guidance. Clear visualization of the mass was rapidly obtained permitting accurate evaluation of size and intracardiac extension (Fig. 2c). Four biopsies were then taken from the right ventricular side under close ICE guidance (Fig. 2d and e; Additional files 3 and 4: Movie 3 and 4). Histopathological analyses showed a poorly differentiated carcinoma characterized by a highly cellular proliferation rate. The pleomorphic epithelioid cells organized in cords of variable thickness resembles dysmorphic hepatocytes with large amounts of cytoplasm. Immunohistochemically studies demonstrate the presence of cytocheratins MNF, Glutamine Syntetase and Heat-shock protein 70, confirming the diagnosis of a metastatic HCC. Successful complete surgical removal of the intracardiac mass confirmed the diagnosis of HCC metastasis.

### Case 3

**Fig. 3:**
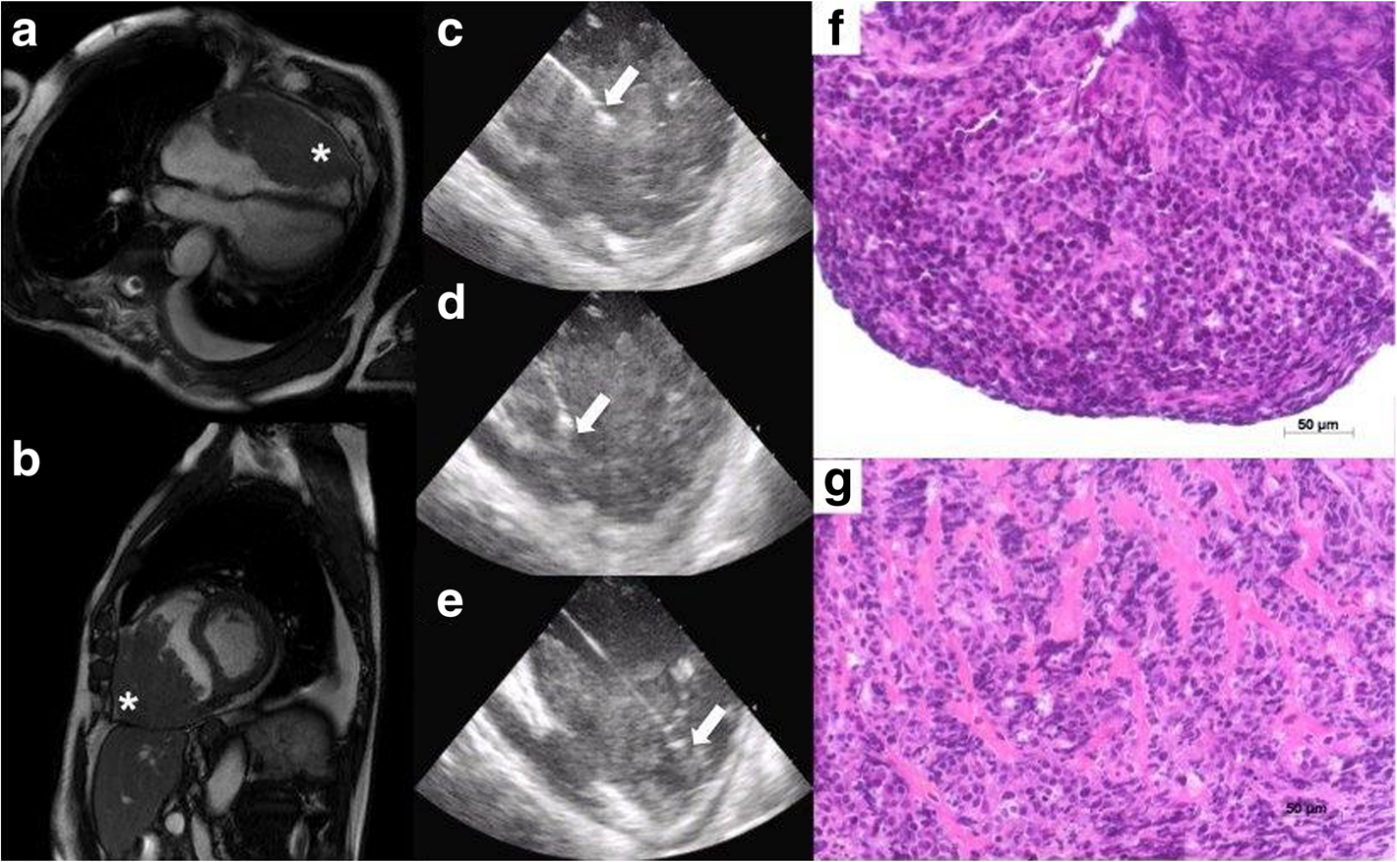
Cardiac magnetic resonance imaging: an irregular voluminous mass (12 × 7.6 cm, thickness of 5.5 cm) extending along the free wall of the RV to the tricuspid annulus is showed in panel (**a** and **b**) (white star). ICE was extremely helpful to identify the heterogeneity of the mass and to selectively place the bioptome, panel (**c**, **d** and **e**) (white arrow). Hystopathological diagnosis revealed a diffuse large B cell non-Hodgkin lymphoma, panel (**f** and **g**)

A 77-years-old male with history of diabetes, myocardial infarction with reduced left ventricular ejection fraction and cerebrovascular disease was referred for an occasional finding of right ventricular mass. TTE showed a dishomogeneous mass that infiltrates the RV from the base to the apex with the only exclusion of the RVOT. CT scanning and CMR highlighted an irregular and frayed voluminous mass of 12 × 7.6 cm with a thickness of 5.5 cm extending along the free wall of the RV to the tricuspid annulus (Fig. 3a and b). The heterogeneity nature of the intracardiac mass permitted to show the importance of ICE guidance in EMB procedures. Indeed, it was possible to selectively guide the bioptome into the different areas of the mass obtaining optimal samples (Fig. 3c-e). Histopathological analyses showed solid sheets and nests of large pleomorphic lymphoid cells, with abundant cytoplasm, conspicuous cleaved nuclei with multiple nucleoli, many atypical mitotic figures and apoptotic cells. Immunohistochemical analyses revealed a B-cell large lymphoma with strong expression of CD20, CD45 but negative for CD10 and CD3. The proliferation rate was approximately 95%, as shown in the Mib1 stain. A fluorescent in situ hybridization (FISH) study showed no evidence of MYC translocation ruling out Burkitt like lymphomas. All these findings confirm the diagnosis of diffuse large B cell lymphoma. After diagnosis the patient was scheduled for a multichemotherapy regimen and is currently under treatment.

## Discussion and conclusions

Intracardiac masses are uncommon findings and can be distinguished as non-neoplastic and neoplastic. Non-neoplastic intracardiac masses include thrombi, pericardial cysts, and prominent anatomic structures. Primary cardiac tumors are rare, occurring with a prevalence ranging from 0.0017 to 0.28% in an autopsy series, while metastatic tumors are more common (2.3–18.3%) [2]. Intracardiac tumors rarely occurs in the RV and usually present with symptoms and manifestations of general malaise, unexplained fever, shortness of breath, right-sided heart failure or syncope. Most primary cardiac tumors are benign. Malignant tumors of the heart are rare and mostly metastatic. Primary cardiac lymphoma is a very rare malignancy, typically non-Hodgkin type with minimal extracardiac involvement [3].

Although clinical diagnosis of intracardiac masses could be mainly done by echocardiography, CT, and/or CMR, histopathological analysis remains crucial for differential diagnosis. Histology provides critical information for treatment and prognosis, which are largely dependent upon tumor histotype and biological behavior. EMB is a valuable tool for diagnosis of intracardiac masses. It is mainly indicated for the investigation of right-sided masses showing an infiltrative or obstructive growth pattern and for the differential diagnosis of sarcomas, lymphomas, and metastatic tumors. Unresectable intracardiac masses may benefit from EMB to plan therapy or palliative treatment [4].

We demonstrated the usefulness of ICE guidance to perform EMB in different sites and pathologies involving the RV. In all cases, the histopathological diagnosis was made on samples obtained by EMB ICE-guided. Our approach was helpful not only for the final diagnosis but also to plan the optimal therapeutic strategy, reserving more invasive procedure only for selected cases. Indeed, the use of ICE eliminates the risks of a diagnostic open-chest procedure. According to this strategy, surgery is reserved only for patients who would benefit the most in terms of prognosis. Another approach to perform transvenous EMB is under fluoroscopic or 2D- or 3D–transesophageal echocardiography (TEE). However, the use of fluoroscopic guidance to perform right ventricular EMB has some drawbacks, in particular the inability to accurately guide the bioptome, the longer radiation exposure and some possible complications including myocardial perforation, tricuspid valve apparatus disruption and arrhythmias. On the other hand, 2D or 3D–TEE can be cumbersome, it requires general anesthesia and a skilled echocardiographist in the operating room [5].

Recently, the use of ICE has increased in many procedures in order to facilitate transseptal puncture, ablation of cardiac arrhythmias, percutaneous mitral valve intervention, interatrial septal abnormalities closure, and left atrial appendage closure. The capability to clearly show intracardiac structures makes ICE a safe and effective tool to guide transcatheter EMB of right side cardiac mass. Moreover, EMB can be performed under conscious sedation with direct visualization of the mass minimizing the risk for perforation. Of note, there are some drawbacks related to the use of ICE, such as the need of a venous approach, the risks of right chamber catheterization and some technical and economical aspects as the probe cost [6].

To the best of our knowledge, there are few reports of ICE-guided EMB of right atrial cardiac tumor [7–9], in contrast we are describing ICE-guided EMB of both metastatic and primary cardiac tumor in the RV for the first time. In all cases, ICE was safely and effectively used to provide precise localization of the mass and of the cardiac structures and to guide EMB. For these reasons, in our experience ICE can be considered an extremely useful tool in EMB of intracardiac mass.

## Additional files

Additional file 1: Movie 1. The whole action of EMB is showed. Fluoroscopy image in RAO view: ICE probe is placed across the tricuspid valve and positioned in the right ventricular inflow tract to image the ventricles in the long axis view. Through a long-deflectable sheath (Agilis, St. Jude Medical, MN, USA) the bioptome is inserted and directed to the mass visualized by ICE. After checking carefully the placement of the bioptome, a sample is taken. (MOV 4285 kb)

Additional file 2: Movie 2. ICE imaging of EMB taken in the right ventricular apex. (MOV 4960 kb)

Additional file 3: Movie 3. ICE imaging of massive multi-lobed intracardiac mass in the right ventricular outflow tract. (MOV 4341 kb)

Additional file 4: Movie 4. EMB guided by ICE. ICE visualizes a good contact between the bioptome and the mass. (MOV 4225 kb)

## Abbreviations

CMR: Cardiac magnetic resonance
EMB: Diagnostic endomyocardial biopsy
HCC: Hepatocellular carcinoma
ICE: Intracardiac echocardiography
RV: Right ventricle
RVOT: Right ventricular outflow tract
TEE: Transesophageal echocardiography
TTE: Transthoracic echocardiography
